# Targeting Pathways in Neuroblastoma: Advances in Treatment Strategies and Clinical Outcomes

**DOI:** 10.3390/ijms26104722

**Published:** 2025-05-15

**Authors:** Diana Benchia, Ovidiu Daniel Bîcă, Ioan Sârbu, Bogdan Savu, Diana Farcaș, Ingrith Miron, Anca Lavinia Postolache, Elena Cojocaru, Olivier Abbo, Carmen Iulia Ciongradi

**Affiliations:** 1 2nd Department of Surgery-Pediatric Surgery and Orthopedics, “Grigore T. Popa” University of Medicine and Pharmacy, 700115 Iasi, Romania; diana.benchia@umfiasi.ro (D.B.); ovidiu-daniel.bica@umfiasi.ro (O.D.B.); bogdan.savu@umfiasi.ro (B.S.); carmen.ciongradi@umfiasi.ro (C.I.C.); 2 Faculty of Medicine, “Grigore T. Popa” University of Medicine and Pharmacy, 700115 Iasi, Romania; miron.diana@d.umfiasi.ro; 3 Department of Mother and Child Medicine-Pediatrics, “Grigore T. Popa” University of Medicine and Pharmacy, 700115 Iasi, Romania; lucmir@gmail.com (I.M.); ancap93@gmail.com (A.L.P.); 4 Department of Morphofunctional Sciences I-Pathology, Faculty of Medicine, “Grigore T. Popa” University of Medicine and Pharmacy, 700115 Iasi, Romania; elena2.cojocaru@umfiasi.ro; 5 Department of Pediatric Surgery, Faculté de Médecine Purpan, Université Toulouse III-Paul Sabatier, Centre Hospitalier Universitaire de Toulouse, 31300 Toulouse, France; abbo.o@chu-toulouse.fr

**Keywords:** neuroblastoma, molecular pathogenesis, tumor microenvironment, precision oncology

## Abstract

Neuroblastoma (NB) is a childhood cancer originating from neural crest cells of the sympathetic nervous system. Despite the advances in multimodal therapy, the treatment of high-risk NB remains challenging. The present review outlines several evidence-related insights into the molecular mechanisms of NB pathogenesis, focusing on genetic drivers (e.g., MYCN amplification) and disrupted signaling pathways (PI3K/Akt/mTOR; Notch; Jak2/STAT3), as well as on the tumor microenvironment’s role in progression and resistance. The authors highlight current and emerging therapeutic strategies, including molecularly targeted agents; immunotherapies; and differentiation approaches under investigation. The complexity and heterogeneity of NB underscores the need for continued translational research and for combined strategies aimed at improving outcomes for affected children, highlighting the need for integration of molecular profiling and precision medicine to guide treatment.

## 1. Introduction

Neuroblastoma (NB) represents a pediatric malignancy originating from neural crest cells that predominantly involves the sympathetic component of the autonomic nervous system, most commonly affecting the adrenal glands and the paraspinal sympathetic ganglia. It is the most common pediatric extracranial solid tumor, mainly affecting children under the age of five, representing 8–10% of all pediatric cancers, and is the third leading cause of malignancy during childhood, behind leukemia and brain tumors [1,2]. NB is a heterogeneous tumor characterized by numerous genetic alterations and deregulated pathways that promote their aggressive behavior and variable response to therapy, although the etiology of NB continues to be poorly understood [3].

NB presents with a complex molecular landscape that has a profound effect on both its pathogenesis and clinical outcomes, with a wide range of clinical behaviors, from spontaneous regression to fatal progression [4]. This heterogeneity has a major impact on patient outcomes and survival [5,6]. Risk stratification, defined as the categorization of patients into low-, intermediate-, and high-risk groups, is the primary determinant of prognosis for NB and assigns patients to treatment protocols based on age at diagnosis, MYCN oncogene amplification, histology, stage of disease, and ploidy status [7,8]. Patients at high risk, typically advanced-stage disease or MYCN amplified, show poor bimodal survival, with long-term survival rates reported as low as 50% [9,10].

On the other hand, low-risk patients may show spontaneous regression or respond favorably to treatment, leading to markedly superior survival [11]. Although improvements in therapeutic strategies have been made, the current treatment regimens are insufficient for patients with high-risk NB. The primary treatment approach typically involves a combination of surgery, chemotherapy, radiation, and immunotherapy [12]. However, challenges in the form of drug resistance and disease relapse persist. NB cells frequently acquire mechanisms that allow them to escape the cytotoxic effects of chemotherapeutics, such as upregulations or modifications in drug transporters, enhanced DNA repair pathways, and apoptotic evasion [13]. Previous studies have shown that relapsed or refractory cases often carry novel genetic profiles or express different biomarkers from the original tumors, which impacts treatment efficacy [14,15,16,17].

This narrative review aims to provide a comprehensive overview of the current knowledge on the molecular landscape of NB, focusing on recent advances in targeted therapies, immunotherapy, and metabolic and differentiation-based strategies designed to combat resistance mechanisms and to improve overall treatment efficacy for NB, highlighting the potential of personalized medicine in ameliorating patient outcomes. The review was started by conducting a search on PubMed, using the keywords “neuroblastoma”, “molecular pathogenesis”, “tumor microenvironment”, “targeted therapies”, and “immunotherapy”, in order to identify relevant publications for inclusion. Publications were chosen for their direct relevance to the focus of the review. The reference lists for each included paper were meticulously reviewed to identify additional publications pertinent to the review’s focus and to enhance the conclusions.

## 2. Molecular Pathogenesis of NB

### 2.1. Genetic and Epigenetic Drivers

#### 2.1.1. Role of MYCN Amplification in Tumor Progression and Poor Prognosis

MYCN is one of the most potent genetic drivers of NB and is commonly amplified in high-risk cases. MYCN is a transcriptional regulator that modulates gene expression in many processes related to cell growth, differentiation, and apoptosis [18]. The MYCN oncogene is found in ~25% of primary NB and ~50% of high-risk cases, representing a hallmark of aggressive disease and unfavorable patient prognosis [19,20]. MYCN amplification is associated with tumor aggressiveness through multiple pathways [21]. Such upregulation of MYCN expression activates pathways related to glycolysis and angiogenesis and promotes an environment that favors a tumorigenic and metastatic process [22].

Additionally, MYCN has been demonstrated to interact with additional oncogenic factors, including hypoxia-inducible factor 1-alpha (HIF-1α), thereby enhancing its influence on tumor progression [22]. The elevated expression of MYCN is typically associated with a more aggressive phenotype, marked by poor differentiation and therapeutic resistance. Furthermore, MYCN demonstrates genomic control that extends beyond simple amplification; various studies indicate that the MYCN gene induces transcriptional addiction in NB, highlighting the essential dependence of these tumor cells on MYCN for survival [23]. Particularly when MYCN expression stays unabated, this addiction makes treatment plans more difficult since tumor cells might avoid regular drugs by means of several resistance mechanisms [23,24]. Along with promoting cell proliferation, elevated levels of MYCN can also drive modifications in apoptosis pathways, enabling cancer cells to persist in unfavorable surroundings [25,26].

In terms of prognosis, patients with MYCN-amplified NB typically experience significantly worse outcomes compared to those with non-amplified tumors. For instance, high-risk NB with MYCN amplification presents with more advanced disease at diagnosis and is linked with a higher proportion of unfavorable histological characteristics, such as poorly differentiated tumors [20,21]. Conversely, the prognostic implications of MYCN status can sometimes be clouded, as occasionally, MYCN non-amplified tumors may still exhibit high levels of MYCN protein, leading to poor outcomes [27]. While MYCN amplification is a well-established hallmark of high-risk NB, the mechanisms underlying its overexpression extend beyond simple gene copy number gain. Several upstream regulators and chromatin dynamics contribute to MYCN dysregulation. One key mechanism involves enhancer hijacking, where distal enhancers are repositioned or activated near the MYCN locus, driving its transcription [28]. Additionally, mutations in chromatin remodeling genes, such as ATRX, ARID1A, or SMARCA4, may lead to epigenetic changes that favor a permissive chromatin state around MYCN [29]. ALK mutations, frequently co-occurring with MYCN amplification, also activate signaling cascades that further enhance MYCN transcription [30]. These multiple layers of control emphasize that MYCN overexpression is not solely the result of genomic amplification, but also a reflection of altered transcriptional and epigenetic regulation in aggressive NB.

In summary, MYCN amplification is a critical genetic alteration in NB that drives tumor progression through various mechanisms, including enhanced proliferation, metabolic reprogramming, and resistance to therapy. Its presence serves as a primary biomarker for risk stratification in NB and is strongly associated with adverse clinical outcomes, underscoring the need for targeted strategies to mitigate its influence in affected patients.

#### 2.1.2. TP53 Mutations and Their Impact on Tumor Suppression and Chemoresistance

The TP53 gene, recognized as a tumor suppressor gene, is essential in cellular responses to stress, including DNA damage, and serves as a key regulator of cell cycle progression and apoptosis [31,32]. The role of TP53 mutations in terms of tumor suppression and chemoresistance in NB is complex and multifaceted, especially considering the low observed TP53 mutation rates in this tumoral context [33]. Although mutations in TP53 are rare in NB, estimated at <2% of primary tumors and ~14% of relapsed disease, the loss of p53 function is still a major issue [34]. In NB, the TP53 gene is normally present but functions poorly, leading to a chemosensitive phenotype and appropriate responses to chemotherapeutic agents, including apoptosis [35]. Alterations in TP53 may result in genomic instability and reduced apoptosis, thereby creating conditions that promote chemoresistance [13,36]. Consequently, functional inactivation of the TP53 pathway, through mutation or other regulatory failures, significantly impacts tumor progression and response to treatment. In NB, alterations impacting the TP53 pathway predominantly consist of genetic and epigenetic modifications within its regulatory network, rather than direct mutations in the TP53 gene itself. The aberrant expression of the MDM2 oncogene, which functions as an E3 ubiquitin ligase that inhibits p53 by facilitating its proteasomal degradation, consequently limiting its tumor-suppressive capabilities, is observed in approximately 36.6% of primary NB cases [13,37]. This overexpression amplifies the effects of pre-existing p53 pathway dysfunction, contributing to elevated chemoresistance as well as to the aggressive clinical course often seen in high-risk NB. Another crucial connection is that between TP53 signaling and the tumor microenvironment (TME). In NB, alterations to the TME may drive adaptive responses that are dependent on TP53 status. The dysfunction of Tp53 could enable escape from immunogenicity and create a pro-tumorigenic microenvironment that is essential for tumor growth and metastasis. Disturbances in functional Tp53 signaling can reduce the metabolic control of the tumor cells and make them more resistant to therapies, as TP53 is critical for the response to chemotherapeutic agents [38].

Overall, although direct mutations in the TP53 gene are uncommon within primary NB, the pathway’s functionality remains significant for understanding both tumor suppression and the development of chemoresistance. Ongoing research indicates that targeting the regulatory elements of the TP53 pathway, particularly using MDM2 inhibitors, offers a promising strategy for enhancing treatment outcomes in NB characterized by impaired TP53 signaling [13,37,39].

#### 2.1.3. The Significance of microRNAs and DNA Methylation in NB Differentiation

Recent studies have drawn great interest to the importance of microRNAs (miRNAs) and DNA methylation in NB differentiation, because of their vital roles in controlling gene expression, cellular behavior, and tumor phenotype [40]. MiRNAs are small, non-coding RNA molecules that regulate gene expression at the post-transcriptional level. Considering that loss of differentiation correlates with tumor aggressiveness, specific miRNAs have been associated with promoting the differentiation of malignantly transformed neuroblasts into mature neuronal cells in NB [41,42]. For instance, Zhao et al. revealed that miR-449a functions as a tumor suppressor in NB by promoting cell differentiation and triggering cell cycle arrest [43]. Moreover, several miRNAs have been revealed to interact with the MYC oncogene family, underscoring their roles in reducing the carcinogenic effects of MYCN commonly observed in NB. This highlights the potential of miRNAs as therapeutic targets, by restoring differentiation processes disrupted in cancer cells [42,43].

DNA methylation, a key epigenetic modification, plays a significant role in regulating gene expression in NB. Aberrant DNA methylation patterns have been correlated with poor differentiation and aggressive tumor behavior [44,45]. For example, Nagase identified a methylator phenotype linked with elevated aggressiveness, indicating that the degree of DNA methylation may serve as a predictor of clinical outcomes [44]. Murphy et al. reported that the oncogenic transcription factor MYCN co-localizes with methyl-binding proteins, such as MeCP2 at hypermethylated DNA regions, highlighting the intricate relationship between transcription factors and DNA methylation in NB [46]. The relationship between miRNAs and DNA methylation illustrates a bidirectional regulatory mechanism that influences NB differentiation. Epigenetic modifications, such as DNA methylation, can silence miRNA expression and may serve as a predictor of clinical outcomes [47]. Notably, retinoic acid, a differentiation agent used in NB therapy, has been shown to modulate both miRNA expression and DNA methylation status, facilitating communication between these two regulatory layers [40]. Both miRNAs and DNA methylation are crucial in the differentiation process of NB. These regulatory mechanisms contribute to the behavior and pathology of NB by affecting the expression and functionality of critical genes involved in neuronal differentiation.

These essential molecular and epigenetic determinants implicated in the etiology of NB are crucial in tumor progression, therapeutic resistance, and the prospective formulation of personalized treatment approaches, based on clinical implication (Table 1).

### 2.2. Key Signaling Pathways in NB Development

#### 2.2.1. Wnt–BMP4–Notch Axis: Influence on Tumor Differentiation and Growth Suppression

This intricate signaling axis of WNT, BMP4, and NOTCH roads suggests a multifaceted regulatory structure in NB, where its interactions significantly influence tumor differentiation and growth suppression [48]. BMP4 within this axis has been demonstrated to mediate mesenchymal–epithelial transition, which may result in growth suppression and increased differentiation of NB cells [49]. The expression of BMP4 is often diminished in poorly differentiated and aggressive NB cases, suggesting its potential role as a tumor suppressor [50]. According to Szemes et al., BMP4 induces the expression of MSX and NOTCH proteins, which are essential for promoting differentiation, and also indicates anti-proliferative effects that could be advantageous for therapeutic applications [50]. The interaction within this signaling axis promotes the transition of tumor cells from a proliferative to a differentiated state, addressing the aggressive nature of high-risk NB. Furthermore, the Wnt pathway exhibits context-dependent effects, where activation can promote differentiation or drive proliferation, depending on the cellular environment and genetic background. For instance, canonical Wnt/β-catenin (β-catenin dependen) signaling has been linked to neuronal differentiation in low-risk NB. However, in high-risk or MYCN-amplified tumors, aberrant Wnt signaling may promote tumor development and stemness [49,51].

#### 2.2.2. PI3K/Akt/mTOR Pathway: Contribution to Cell Survival and Therapy Resistance

The PI3K/Akt/mTOR signaling pathway plays a vital role in regulating cell survival, growth, and metabolism, making it a key player in NB progression and chemoresistance. The activation of this pathway is common in NB tumors, frequently associated with poor prognosis and treatment resistance [52]. The mTOR complex integrates signals from growth factors and nutrients, thereby facilitating cellular responses that promote tumor survival and growth [49]. The PI3K/Akt/mTOR pathway in NB increases the expression of pro-survival and anti-apoptotic proteins, inhibiting programmed cell death and enabling malignant cells to thrive despite chemotherapeutic agents [52]. Notably, NB cells with anaplastic lymphoma kinase (ALK) mutations frequently depend on this pathway for growth and tend to exhibit considerable resistance to therapies targeting various mechanisms, highlighting the clinical challenges associated with this signaling network [53]. Inhibitors targeting components of the PI3K/Akt/mTOR pathway have shown promise in preclinical studies, suggesting that combinatorial strategies utilizing these inhibitors might improve treatment outcomes in NB cases resistant to standard therapies [54,55].

#### 2.2.3. Jak2/Stat3 Signaling: Effects on Tumor Proliferation and Potential for Therapeutic Inhibition

The role of Jak2/Stat3 signaling in NB has been widely investigated, with evidence demonstrating that this pathway promotes tumor cell growth and apoptosis resistance [56]. The activation of Stat3 is frequently observed in high-risk NB, promoting cellular proliferation and survival, which contributes to the aggressive nature of the disease [57]. This pathway is often activated by cytokines such as IL-6, commonly elevated in the NB microenvironment [57]. The inhibition of the Jak2/Stat3 signaling pathway represents a promising therapeutic approach for NB, as it not only blocks survival signals but also interferes with mechanisms that promote tumor invasion and metastasis [57,58]. Therapeutic agents that inhibit this signaling pathway have demonstrated potential in decreasing tumor growth and enhancing the efficacy of existing treatments, indicating their viability as novel therapeutic strategies in NB management.

A complete understanding of the molecular mechanisms underlying these interconnected signaling pathways offers promising pathways for the development of targeted therapeutic strategies (Table 2).

## 3. TME and Its Role in NB Progression

TME in NB is a multifaceted entity composed of various cellular and extracellular components that work synergistically to promote tumor progression, increase metastatic capabilities, and evade immune surveillance [59,60,61].

### 3.1. Interaction Between NB Cells and Stromal Components

The interaction between NB cells and stromal components significantly influences the TME, affecting tumor behavior and treatment outcomes. The TME consists of various cell types, including cancer-associated fibroblasts (CAFs), endothelial cells, and immune cells, which create a supportive niche for tumor growth [62,63]. CAFs, in particular, have been associated with tumor progression by providing structural components and secreting growth factors and cytokines that promote NB cell proliferation and survival [64]. These interactions create an environment that not only facilitates tumor growth but also promotes resistance to standard therapies, thus complicating treatment efforts [63]. A noteworthy aspect of the TME in NB is the ability of stromal cells to help cancer cells evade immune surveillance. The stromal components can inhibit effective anti-tumor immune responses by secreting immunosuppressive factors, including transforming growth factor-beta (TGF-β) [65,66]. The ongoing interaction between NB cells and stromal components influences the tumor’s developmental path and contributes to its aggressiveness and resistance to therapy [62].

### 3.2. Role of Immune Cells and Inflammatory Cytokines in Tumor Development

Immune cells play a critical role in the NB microenvironment, significantly influencing tumor development and progression. The speed of tumor proliferation is dependent on the interaction between malignant cells and the host’s immune system. Various immune cell populations, including macrophages, natural killer (NK) cells, and cytotoxic T lymphocytes, infiltrate NB tissues, and their activities can either suppress or enhance tumor growth depending on their functional status [67]. For instance, macrophages often undergo polarization, adopting either pro-tumorigenic (M2) or anti-tumorigenic (M1) phenotypes. Notably, the increased presence of M2 macrophages has been associated with advanced disease and poorer outcomes, as these cells secrete inflammatory cytokines like IL-6 and IL-10, contributing to immune evasion [68]. Additionally, the NB TME often exhibits a distinct inflammatory profile characterized by elevated levels of inflammatory cytokines, such as IL-1β and TNF-α [68]. These cytokines can lead to metabolic changes in cancer cells, further enhancing their growth and survival capabilities [68,69]. The interplay between these immune cells and the NB cells suggests that modulating the immune landscape could serve as a viable therapeutic strategy to disrupt tumor growth and improve patient outcomes [70].

### 3.3. The Influence of Hypoxia on Tumor Aggressiveness and Metastasis

One crucial feature of the TME is its hypoxic nature, which profoundly impacts NB progression. Rapid tumor cell growth raises the need for oxygen supply, which the surrounding blood vessels are unable to provide, limiting the cells’ access to oxygen and causing hypoxia [71]. HIF-1α is often upregulated in the hypoxic conditions typical of NB, which facilitates tumor cell survival and promotes angiogenesis [72]. The interplay between hypoxic tumor cells and the surrounding stroma can lead to a more aggressive phenotype, making the tumor less responsive to therapies [59]. Moreover, in NB, hypoxia has been linked to increased invasion and metastasis, as it triggers pathways that promote cell motility, epithelial–mesenchymal transition (EMT), and angiogenesis [73,74]. Studies have indicated that preconditioning NB cells under hypoxic conditions enhances their capacity to metastasize, leading to a more aggressive tumor phenotype [73]. The expression of genes associated with vascularization and metastasis, such as matrix metalloproteinases (MMPs) and vascular endothelial growth factor (VEGF), tends to increase under hypoxic stress, fostering a microenvironment conducive to tumor spread [73,75]. This chronic hypoxic exposure may further complicate treatment regimens by inducing radio-resistance and reducing the effectiveness of chemotherapeutic agents [69].

The intricate interactions between NB cells and stromal components, the engagement of immune cells along with inflammatory cytokines, and the effects of hypoxia all contribute substantially to tumor development, progression, and therapeutic resistance (Table 3). Understanding these relationships enhances the potential for developing targeted therapies aimed at improving outcomes in NB patients.

## 4. Advances in NB Therapy

Platinum-based chemotherapeutic agents, such as cisplatin and carboplatin, are frequently used in high-risk NB treatment regimens. These agents exert their cytotoxic effects primarily through DNA cross-linking, which leads to double-strand breaks and the activation of p53-dependent apoptotic pathways. Therefore, the functional integrity of the p53 pathway is critical to treatment response [76,77,78,79]. As discussed in Section 2.1.2, alterations in TP53 or disruptions in p53 regulatory proteins, such as MDM2 or CHK1, may lead to impaired apoptosis and chemoresistance [13,24]. This mechanistic link underscores the importance of the p53 pathway status in shaping both prognosis and therapeutic response to DNA-damaging agents in NB.

### 4.1. Targeted Molecular Therapies

#### 4.1.1. PI3K and mTOR Inhibitors in Overcoming Chemoresistance

The mTOR and PI3K pathways serve as essential regulators of metabolic activities, survival, and cell proliferation, as previously noted. Research indicates that NB cells may develop resistance to chemotherapeutic agents through the activation of the PI3K/Akt/mTOR pathway, which makes it a desirable target for therapeutic intervention [67]. Inhibitors like rapamycin and other class-specific mTOR inhibitors have shown the capacity to sensitize NB cells to routinely used treatments, such as cisplatin and doxorubicin [68]. These inhibitors may help to overcome chemoresistance by downregulating the mTOR pathway, which lowers cell viability and induces death in resistant NB cell lines. Furthermore, combining these inhibitors with standard chemotherapeutics could increase effectiveness and raise the likelihood of obtaining long-lasting responses in high-risk NB patients [69].

#### 4.1.2. Inhibitors of Histone Deacetylases (HDAC) and Their Epigenetic Impact

Histone deacetylases (HDACs) are key regulators of gene expression by removing acetyl groups from histones, resulting in condensed chromatin and transcriptional repression. Abnormal HDAC activity in NB has been connected to chemoresistance as well as the ongoing presence of tumorigenic characteristics [80]. For restoring acetylation levels, some HDAC inhibitors, including vorinostat and panobinostat, have shown promise in reactivating silent tumor suppressor genes and improving apoptosis [81,82]. Studies indicate that, in addition to causing apoptosis, HDAC inhibitors might cause NB cell differentiation, hence offering a dual therapeutic strategy [80]. HDAC inhibitors could shift the balance away from undifferentiated tumor states toward more mature neuronal phenotypes by targeting the acetylation status of histones and hence altering gene expression profiles, which is good for patient prognosis. Importantly, ongoing clinical trials are evaluating the effectiveness of these drugs both as monotherapies and in combination with other treatments, hence demonstrating their significance in NB care [83].

#### 4.1.3. Targeting the Notch Pathway: Potential for Growth Arrest and Differentiation

The Notch signaling pathway is essential for the regulation of cell fate decisions, proliferation, and differentiation. Abnormal Notch signaling in NB has been linked to malignancy by promoting cell survival and proliferation [84]. It has been demonstrated that blocking this pathway, especially by blocking Notch ligands like Delta-like 1 (DLL1), may be able to stop the evolution of NB [84]. Studies show that blocking Notch signaling causes NB cells to stop growing and promotes their differentiation, hence integrating differentiation therapy into treatment strategies [85]. Among the several treatment approaches aimed at Notch signaling now being researched are γ-secretase inhibitors blocking downstream signaling activation [82]. Experimental data suggest that Notch inhibition is a possible path for targeted therapy since it can significantly lower NB cell viability and improve the effectiveness of standard treatments. The possibility to affect tumor differentiation and control growth dynamics offers a strong justification for creating Notch-targeted treatments in the setting of NB [86].

### 4.2. Immunotherapy and Novel Treatment Strategies

#### 4.2.1. Antibody-Based Therapies Targeting GD2 and ALK

Immunotherapy for high-risk NB has become increasingly reliant on antibody-based therapies, notably those that target disialoganglioside GD2. Terzić et al. found that incorporating dinutuximab, an anti-GD2 monoclonal antibody, into multimodal treatment regimens led to improved survival outcomes for patients [87]. A marker usually overexpressed in NB compared to normal tissues, dinutuximab allows the immune system to identify and kill NB cells expressing GD2 on their surface [88]. Studies indicate that its effectiveness is improved by combining anti-GD2 therapy with other therapies including differentiation agents or cytokine therapy (IL-2 and GM-CSF) [89]. Still, issues concerning clinical relevance persist since a large percentage of patients continue to relapse because of treatment resistance linked to reduced GD2 expression or other compensatory mechanisms [90,91,92]. Apart from GD2-targeting strategies, ALK inhibitors have been investigated especially for NB patients with ALK mutations. In clinical environments for patients with relapsed or refractory NB, ALK inhibitors like crizotinib and ceritinib have shown potential, hence proving notable beneficial effects against cancer [84]. Knowing the suitable setting for the usage of ALK inhibitors together with GD2-directed treatments could help to open more successful treatment plans for high-risk NB.

Recent clinical trials have continued to explore and optimize anti-GD2 therapies in NB, both as monotherapies and in combination with other agents, such as IL-2 or IL-15 (Table 4).

#### 4.2.2. The Role of Immune Checkpoint Inhibitors in NB Management

Immune checkpoint inhibitors have received interest in NB treatment because they help to attenuate the immunosuppressive tumor microenvironment that is characteristic of this malignancy. By restoring T-cell activity against NB cells, agents aiming at the programmed cell death protein 1 (PD-1) and its ligand PD-L1 could possibly improve the efficacy of current treatments [97]. Preclinical studies indicate that anti-GD2 antibodies combined with PD-1 inhibitors might work together to enhance antitumor immune responses [16]. The effectiveness of checkpoint inhibitors is significantly impaired, however, by the natural immunosuppressive mechanisms in the NB tumor microenvironment [98]. NB cells, for example, frequently produce several immunological checkpoint proteins that could compromise T-cell activation and function [98]. Ongoing clinical studies assessing the use of checkpoint inhibitors together with conventional treatments will determine their efficacy, which might improve survival rates in high-risk groups [16].

#### 4.2.3. Emerging Role of Chimeric Antigen Receptor (CAR) T-Cell Therapy in High-Risk NB

CAR T-cell therapy is a new and revolutionary way to treat high-risk NB. In order to use the immune system’s ability to eradicate tumors, scientists are genetically modifying T cells to produce CARs that specifically target antigens on NB cells [99]. Early-phase clinical studies have shown promise for CAR-T cells targeting GD2, with positive preliminary findings on safety and effectiveness [99,100]. One of the key advantages of CAR-T treatment is its ability to treat tumors with low levels of MHC-I expression, which frequently interfere with standard T-cell responses [101]. The possibility of including CAR-T therapies, especially those that additionally target several antigens or use new immunomodulatory techniques, may offer notable gains in patient outcomes as our knowledge of NB biology develops. For example, recent studies combining CAR-T cell treatment with checkpoint inhibitors aim to improve T-cell persistence and functionality in the tumor microenvironment [102,103].

Despite the encouraging preclinical and early clinical outcomes of CAR-T cell therapy in NB, several barriers limit its effective translation into routine clinical use, particularly in solid tumors. A major obstacle is the immunosuppressive TME, which actively inhibits T-cell function through the presence of regulatory T cells, myeloid-derived suppressor cells (MDSCs), and immunosuppressive cytokines, such as TGF-β and IL-10 [104]. In addition, antigen heterogeneity and antigen loss can lead to immune escape, especially in tumors where GD2 expression is variable or downregulated following treatment [95,105]. Another major challenge is T-cell exhaustion, a state characterized by reduced proliferation and cytokine production due to chronic antigen exposure, often driven by persistent CAR signaling or interaction with checkpoint molecules like PD-1/PD-L1 [106]. Overcoming these obstacles may require combination approaches, such as CAR-T engineering with checkpoint resistance (e.g., PD-1 dominant-negative receptors), the co-expression of cytokines like IL-15, and the targeted disruption of stromal components to facilitate better tumor access and function [93,107].

### 4.3. Differentiation Therapy and Metabolic Reprogramming

#### 4.3.1. Retinoic Acid-Induced Differentiation and Its Clinical Impact

Retinoic acid, a vitamin A derivative, has been a key component of differentiation therapy for NB, particularly in high-risk cases. Its mechanism involves the differentiation of NB cells into more mature neuronal-like cells by means of gene expression modulation via retinoic acid receptors (RARs) and retinoid X receptors (RXRs) [108]. This approach increases the sensitivity of these cells to chemotherapeutic drugs and lowers the aggressiveness of tumors. Clinical trials have shown that introducing all-trans retinoic acid (ATRA) to conventional treatment protocols can raise event-free and general survival rates in NB patients [109]. Specifically, ATRA causes growth arrest and stimulates differentiation, usually resulting in the end of cell proliferation in tumor cells, which is a key element for controlling high-risk NB cases [110]. The successful integration of ATRA into therapeutic strategies shows the potential of differentiation therapy as a powerful tool to improve NB treatment results, especially for patients with high-risk characteristics.

#### 4.3.2. Role of Metabolic Vulnerabilities in Therapeutic Targeting

Metabolic reprogramming is a key feature of NB, particularly in MYCN-amplified instances, where changed nutrition usage patterns create unique vulnerabilities that can be exploited therapeutically. Studies show that NB cells show more dependence on particular metabolic pathways like glutamine metabolism and the serine–glycine–one-carbon (SGOC) cycle [111,112]. Focusing on these pathways, especially in situations resistant to traditional chemotherapy, might be a successful approach for managing NB. For example, MYCN amplification has been connected to greater glycolytic activity and a higher need for serine and glycine, suggesting that blocking pathways linked to these amino acids could cause metabolic stress and death in NB cells [113,114]. While preserving normal tissues, this susceptibility to metabolic disturbances offers a chance to create new therapeutic approaches using particular inhibitors targeting metabolic dependencies and causing apoptosis specifically in cancer cells [115,116]. Moreover, the investigation of the interaction between metabolic pathways and the tumor microenvironment exposes more complexity that could guide therapy choices [117].

The progression towards more individualized and efficacious therapies in high-risk and recurrent cases is characterized by novel therapeutic techniques, grounded on targeted molecular approaches, immunotherapeutic modalities, and treatments based on metabolic or differentiation principles (Table 5).

## 5. Future Directions and Unanswered Questions in NB Treatment

While there have been significant advances in genomic precision medicine for adult cancers, numerous challenges persist in the field of pediatric oncology. The genetic characteristics of pediatric tumors differ markedly from those of adult tumors, necessitating extensive research to explore the unique aspects of pediatric tumors [118]. Despite tremendous progress, many unsolved questions and obstacles remain. At present, research findings regarding the treatment of NB remain inconsistent, and ongoing advances are being made in combination therapies, targeting established treatment objectives [119,120]. Precision oncology, personalized medicine, and innovative therapeutic approaches are all part of the future of NB treatment [121]. Furthermore, emerging therapeutic strategies should not only aim to increase survival but also to minimize long-term toxicity, with particular focus on preserving fertility and overall quality of life in pediatric patients [122,123,124,125,126,127,128,129,130].

### 5.1. The Need for Personalized Medicine Based on Genetic Profiling

Particularly in the context of NB, personalized medicine emphasizes the need for genetic analysis to customize treatment plans to fit specific patient requirements. Advances in genomics have made it possible to find particular genetic changes linked to NB, such as MYCN amplification and ALK mutations, which could suggest different prognostic results [131,132]. Genetic profiling allows precise risk classification, hence helping doctors to classify patients into low-, moderate-, or high-risk categories, which then guides the treatment selection [132]. Moreover, including molecular markers in therapeutic decision-making helps to maximize the use of targeted medicines. Technological developments, such as next-generation sequencing, have increased our knowledge of the mutational terrain of NB, hence highlighting the complexity of its biology and the necessity of tailored treatment strategies [133]. This implies the possibility of better patient outcomes by means of customized treatments, directly targeting the unique genetic composition of every tumor.

### 5.2. Advances in Precision Oncology and Combination Therapies

Precision oncology aims to improve therapy effectiveness and reduce needless toxicity by means of recognizing and addressing molecular changes particular to each tumor. Recent developments include the development and application of targeted treatments such as ALK inhibitors and anti-GD2 antibody therapy [131]. NB treatment is also seeing increasing interest from combination therapies that mix molecularly targeted medicines with conventional chemotherapy and immunotherapy. Aiming to harness synergistic benefits amongst treatments, these combination approaches tackle the multidimensional character of NB and its adaptive resistance mechanisms [134]. The simultaneous use of immune checkpoint inhibitors with immunotherapies targeting GD2, for example, has shown promise since these combinations could improve anti-tumor immune responses and lower immune evasion [70]. Translating early preclinical results into successful clinical practice will require more research on the pharmacodynamics and best sequencing of these combined treatments.

### 5.3. Challenges in Translating Preclinical Findings into Clinical Practice

While the preclinical findings are promising, translating these insights into effective therapeutic treatments for NB poses significant challenges. One significant problem is the absence of strong patient-derived models accurately reflecting tumor heterogeneity, which complicates the assessment of the effectiveness of new treatments prior to clinical trials [135]. The standardization of treatment procedures and responses is further hampered by genetic and epigenetic profile variation across NB cases. Furthermore, the immune dynamics inside the TME may significantly vary across preclinical models and actual patients. Therapies that seem successful in vitro or in early in vivo models, therefore, could not produce the same favorable results in larger clinical trials [70]. Moreover, the complexity of pediatric tumors calls for a concentrated drug development strategy taking into account age-specific biological reactions, since usual adult cancer treatments might not be appropriate. Dealing with these difficulties calls for further funding in translational research, including the creation of predictive biomarkers that can guide treatment choices and raise the probability of favorable results [136,137].

### 5.4. Collaborative Infrastructures and Biomarker Validation

The development of effective CAR-T cell therapies for NB depends increasingly on multi-omics consortia and cross-institutional collaboration. These consortia integrate genomic, transcriptomic, epigenomic, and proteomic profiling to define tumor heterogeneity, identify predictive biomarkers, and guide antigen selection [138]. Initiatives such as the Pediatric MATCH trial and the Children’s Oncology Group (COG) provide platforms for genomic and transcriptomic analyses, facilitating the identification of novel CAR targets and resistance mechanisms [139,140]. These collaborations enable the development of standardized biomarker validation strategies, including single-cell RNA sequencing and high-parameter flow cytometry, to monitor CAR-T cell persistence and tumor antigen expression during treatment. Linking these molecular platforms with centralized biobanks and data-sharing networks enhances the consistency of correlative studies across institutions, addressing the challenges posed by tumor heterogeneity and immune evasion in pediatric solid tumors [141].

## 6. Conclusions

NB remains one of the most challenging pediatric malignancies due to its molecular complexity and clinical heterogeneity. Understanding the intricate molecular pathogenesis of NB is essential for identifying new therapeutic targets and guiding the development of more effective treatment strategies. The ongoing advances in precision medicine have established an effective foundation for innovation and validation in NB, facilitating its future development. While combination therapies might improve treatment effectiveness, advances in genetic profiling are the key to customizing treatments for individual patients.

This review emphasizes the important advances achieved in clarifying the genetic and epigenetic factors influencing NB, including MYCN amplification, TP53 dysfunction, and alterations in signaling pathways. Despite these advances, the translation of molecular insights into effective clinical interventions continues to pose challenges, especially in high-risk and relapsed cases. Ongoing advances in genomic profiling, immunotherapy, and differentiation-based therapies are defining a new era in the management of NB. Nevertheless, there are still many obstacles to overcome before preclinical findings can be applied in clinical settings, which calls for more research and cooperation from all parties involved. Future progress requires integrated collaboration among researchers, clinicians, and pharmaceutical developers to create innovative treatments that target both tumor cells and their supportive microenvironment, ultimately improving prognosis and quality of life for affected children.

## Figures and Tables

**Table 1:** Genetic and epigenetic drivers involved in the pathogenesis of NB.

Molecular Factor	Mechanism of Action	Clinical Implications	References
MYCN	- Amplification → transcriptional activation of pro-proliferative, anti-apoptotic, angiogenesis, and HIF-1α genes - Metabolic reprogramming	- Major risk marker - Associated with severe prognosis and resistance to treatment	[18,20,22,23,24,25,26]
TP53	- Loss of function by inhibition (e.g., MDM2 ↑), less commonly by mutation	- Genomic instability - Decreased apoptosis - Increased resistance	[13,33,34,35,36,38]
microRNAs (e.g., miR-449a)	- Regulation of gene expression post-transcriptionally - Induces differentiation and cell cycle arrest	- Therapeutic potential in restoring tumor cell differentiation	[40,41,42]
DNA methylation	- Hypermethylation → repression of suppressor genes and miRNAs	- Aggressive phenotype - Possible biomarkers for prognosis and treatment	[40,44,45,46,47]

**Table 2:** Key Signaling Pathways in NB Development.

Pathway	Mechanism of Action	Clinical Relevance	Targeted Therapeutic Strategies	References
Wnt–BMP4–Notch Axis	- Regulates mesenchymal–epithelial transition; promotes cell differentiation; and suppresses proliferation	- Loss of BMP4 associated with aggressive, poorly differentiated tumors	- Therapies enhancing BMP4 signaling or modulating Wnt/Notch balance	[48,49,50]
PI3K/Akt/mTOR Pathway	- Promotes cell survival, proliferation, and metabolism	- Therapy resistance and poor prognosis	- PI3K/mTOR inhibitors - Combination therapies	[50,52,54]
Jak2/Stat3 Pathway	- Stimulates tumor proliferation - Inhibits apoptosis	- Tumor aggressiveness	- Jak2/Stat3 inhibitors	[57,58]

**Table 3:** Tumor Microenvironment in NB—Key Elements and Effects.

Component	Key Roles in NB	Impact on Tumor Progression	References
**Stromal cells (e.g., CAFs and endothelial cells)**	- Structural support - Growth factors and cytokines	- Tumor progression - Support immune evasion via TGF-β secretion - Therapy resistance	[60,62,64]
**Immune cells (macrophages, NK, and T cells)**	- Pro-tumorigenic (M2) macrophage phenotype secretes IL-6 and IL-10	- Promote immune escape and tumor survival	[66,67]
**Inflammatory cytokines (IL-1β and TNF-α)**	- Induce metabolic reprogramming - Promote cell survival	- Increase cell proliferation and survival	[67,68,69,70]
**Hypoxia and HIF-1α activation**	- Facilitates tumor cell survival - Promotes angiogenesis - Increased invasion and metastasis - Upregulates VEGF and MMPs	- Aggressive tumor phenotype - Increased invasiveness - Therapy resistance	[59,71,73,74]

**Table 4:** Comparative results of combined anti-GD2 antibody treatment with other agents versus monotherapy in NB: recent clinical trials.

Trial/References	Treatment Arm	Patient Population	Response Rate (ORR) CR/PR	Event-Free Survival (EFS)	Overall Survival (OS)	Key Findings
NCT03373097 [93]	GD2-targeted CAR-T cells with inducible suicide gene	Relapsed/refractory high-risk NB	63% (33% CR)	36% at 3 years	60% at 3 years	Demonstrated feasibility and sustained antitumor effect with manageable safety profile
NCT02743429 [94]	Dinutuximab beta long-term infusion (LTI) monotherapy	Relapsed/refractory stage 4 NB	26% (CR/PR) at 24 weeks	31% at 3 years	66% at 3 years	DB-LTI is an active and tolerable therapeutic option
NCT03294954 [95]	GD2-CAR-NKT cells co-expressing IL-15	Relapsed/refractory NB	25% (1 CR, 2 PR)	Not reported	Not reported	Safe with no dose-limiting toxicities; IL-15 may enhance CAR-NKT cell activity
NCT01711554 [96]	Dinutuximab beta ± low-dose IL-2 post-haploidentical SCT	Relapsed high-risk NB	Not specified	Not specified	Not specified	Combining IL-2 with anti-GD2 post-transplant is feasible; further studies needed to assess efficacy

CR—complete response; PR—partial response.

**Table 5:** Advances in NB Therapy.

Therapeutic Strategy	Target/Mechanism	Key Agents	Clinical Insight	Survival Data (EFS/OS)	References
**PI3K/mTOR inhibitors**	PI3K/Akt/mTOR pathway	Rapamycin and Temsirolimus	Enhances response to standard chemotherapy; synergistic with cisplatin	Data not yet mature; early-phase trials	[67,68,69]
**HDAC inhibitors**	Histone deacetylation/epigenetic reprogramming	Vorinostat and Panobinostat	Induces apoptosis and differentiation; under clinical investigation	No EFS/OS published to date	[80,81,82]
**Notch pathway inhibition**	Inhibits DLL1-Notch signaling	γ-secretase inhibitors	Reduces proliferation; preclinical benefit shown	Preclinical; no survival data yet	[84,85,86]
**Anti-GD2 antibody therapy**	GD2 targeting; immune activation	Dinutuximab + IL-2/GM-CSF	FDA-approved; part of standard care; and enhances NK-mediated killing	EFS ~66%, OS ~86% at 2 years	[87,88,89,91]
**ALK inhibitors**	Targets ALK mutations	Crizotinib, Ceritinib	Effective in relapsed ALK-mutant cases	ORR ~36%; OS data limited in small trials	[92]
**Checkpoint inhibitors**	Blocks PD-1/PD-L1 axis	Nivolumab, and Pembrolizumab	Limited alone; being tested with anti-GD2 or CAR-T	No meaningful OS benefit alone in NB	[16,91,97]
**CAR-T cell therapy**	Engineered GD2-directed T cells	GD2-CAR-T (e.g., GD2-CART01)	High response in relapsed NB; IL-15 and suicide switch improve safety	ORR 63%, 3-yr EFS 36%, OS 60%	[99,100,101,102,103]
**Retinoic acid therapy**	RAR/RXR signaling → cell differentiation	13-cis-RA (ATRA)	Part of post-consolidation therapy; improves outcomes in high-risk NB	3-yr EFS improved post-induction; OS benefit modest	[108,109,110]
**Metabolic targeting**	Blocks MYCN-related metabolism (glutamine and serine)	CB-839 and PHGDH inhibitors	Tumor-selective; especially effective in MYCN-amplified NB	Preclinical; no survival data in humans	[111,112,113,114,116]

OS—Overall survival; EFS—Event free survival; and ORR—objective response rate.
